# Metallurgical Aspects of Ni-Coating and High Temperature Treatments for FBG Spectrum Regeneration

**DOI:** 10.3390/ma16082943

**Published:** 2023-04-07

**Authors:** Carla Lupi, Cristian Vendittozzi, Erwin Ciro, Ferdinando Felli, Daniela Pilone

**Affiliations:** 1 Dipartimento Ingegneria Chimica Materiali Ambiente, Sapienza Rome University, Via Eudossiana 18, 00184 Rome, Italy; 2 Campus FGA-UnB, Universidade de Brasília, Brasília 72444-240, Gama Brasília-DF, Brazil; 3 Department of Engineering Sciences, Università Degli Studi Guglielmo Marconi, 00193 Rome, Italy

**Keywords:** nickel electrodeposition, FBG coating, high-temperature treatments, coating characterization, FBG regeneration

## Abstract

The structural integrity of mechanical components is assessed by FBG sensors in many industrial fields. The FBG sensor has a relevant application at very high or low temperatures. To avoid the variability of the reflected spectrum and the mechanical properties degradation of the FBG sensor, metal coatings have been used to guarantee the grating’s integrity in extreme temperature environments. Particularly, at high temperatures, Ni could be a suitable selection as a coating to improve the features of FBG sensors. Furthermore, it was demonstrated that Ni coating and high-temperature treatments can recover a broken, seemingly unusable sensor. In this work, two main objectives were pursued: first, the determination of the best operative parameters to achieve the most compact, adherent, and homogeneous coating; second, the correlation between the obtained morphology and structure and the FBG spectrum modification, once Ni was deposited on the FBG sensor. The Ni coating was deposited from aqueous solutions. By performing heat treatments of the Ni-coated FBG sensor, it was investigated how the wavelength (WL) varied as a function of temperature and how that variation was caused by the structural or dimensional change of the Ni coating.

## 1. Introduction

Monitoring continuously, accurately, and in real time, the operating environment of engineering systems is of paramount importance. Environmental parameters such as temperature, pressure, and humidity can vary in the operating environment, affecting the performance of engineering systems to the point of jeopardizing their operation. The parameter that is of greatest interest because it most influences the operation of equipment and structures is temperature, especially when it reaches very high or very low levels, such as cryogenic temperatures. In this case, the operating environment is particularly hostile to most measurement systems and related sensors. 

Traditional electrical sensors (such as thermocouples) are usually used to monitor a wide temperature range from room temperature to high-temperature in harsh environments and extreme working conditions. However, the use of electrical sensors under these conditions is constrained by their susceptibility to electromagnetic interference and the electrical and thermal conductivity of the wires, which can generate magnetic fields and heat. 

A good alternative is fiber-optic Bragg grating (FBG) sensors [1]. FBGs have many advantages over their electrical counterparts, offering small physical size, low weight, electrically passive operation, immunity to electromagnetic interference (EMI), and corrosion resistance, and they are suitable for being attached to or embedded into structures. Furthermore, FBGs have high-temperature sensitivity and good temperature resistance, but common bare FBGs, specifically those that are used as temperature sensors, rather than as strain gauges, have a rather limited operating temperature range, from −20 °C to +70 °C. However, the high-temperature, corrosive chemicals, and high mechanical stress often encountered in harsh environments impose serious challenges on the SiO_2_ fiber. 

In order to protect the FBG, a protective layer often needs to be added to the surface of the fiber optic. Typically, the FBG is protected with a polymer that coats the fiber, such as acrylate, polyimide, Teflon, PMMA, PEEK, and Ormocer [2]. However, most of them are viscoelastic materials, which would burn or melt at temperatures above 300 °C. 

Even more remarkable is the temperature-dependent modification of the reflected spectrum. The specific wavelength reflected by the grating (*λ_B_*) depends on the spatial period of the modulation of the refractive index forming the grating (*Λ*), following the Bragg condition, that is:

(1) $$\lambda_{B}=2n_{eff}\Lambda$$

where *n_eff_* is the effective refractive index of the fiber [2]. The Δ*λ_B_* shift of the central wavelength (CWL) *λ_B_* depends on temperature. The shift of the CWL due to temperature variations is given by:

(2) $$\Delta \lambda_{B,T}=2\left(\Lambda \frac{\partial n}{\partial T}+n\frac{\partial \Lambda }{\partial T}\right)\Delta T$$

where (∂Λ/∂T) 1/Λ is the thermal expansion coefficient and $\left(\partial n_{eff}/\partial T\right)1/n_{eff}$ is the FBG thermo-optical coefficient [3].

For conventional UV-inscribed FBGs, the reflected spectrum of the sensor does not change significantly from room temperature to 400 °C, above which the grating loses its excellent sensing performances [4]. The gratings written in the boron-germanium-silicon fiber, on the other hand, resist up to 400 °C [5,6], while direct-writing gratings resist up to 950 °C [7]. Despite the performance achieved with the latter type of sensors, the goal is to protect the more conventional boron-germanium-doped SiO_2_ fiber grating mechanically and thermally so that they can operate stably and with good sensitivity at least up to 800 °C. FBG sensing is an established technology that offers a monitoring solution in harsh conditions where electrical sensors typically have difficulty providing reliable results [8]. The latest manufacturing technologies for FBGs offer sensors on thinner fibers than traditional 9/125, and the ability to inscribe gratings without the need to remove the polymer coating, in order to avoid weakening the fiber due to stripping. These sensors have already been shown to withstand applications above 800 °C [9], despite this, the need to produce a metal coating becomes newly significant when the aim is to combine better mechanical protection (compared to polymer coatings) to facilitate handling, with enhanced sensitivity under extreme temperatures [10,11], and even more when the sensor is to be embedded within a metal matrix. Compared to surface-bonded sensors, embedded sensors can provide punctual information at certain critical points from inside the structure in real-time [12,13]. In this case, the absence of a coating, or the presence of a polymer coating, reduces the adhesion with the SiO_2_ fiber and worsens the performance of the grating, causing an inhomogeneity in the transfer function of the monitored magnitude, whether one wants to insert the sensor directly into a cast matrix (this is the case with low melting metals such as copper), or encapsulate the sensor in a pre-crafted groove, inside material [14].

Here, metallization is proposed to protect the FBG from mechanical and thermal stresses. Currently, many techniques have been considered to achieve metallic coating of fibers, such as casting [15], chemical or physical vapor deposition [16,17,18,19], electroless chemical plating [20,21,22], and electrodeposition [23,24]. Among all the above methods, electrodeposition is probably the most suitable technique because it is simple, cost-effective, can be performed at room temperature to avoid thermal deformation, and it allows the deposition of large amounts of metal. This paper proposes a method in which a preliminary treatment of the fiber is carried out to provide it with a conductive layer, and then the protective metallic layer is deposited by electrodeposition. 

The metallic coatings seem to have better performance in terms of both resistance to high-temperature and mechanical resistance. For low-temperature applications, the high coefficient of linear thermal expansion is exploited to amplify the signals. In our study, the Ni coating was selected for performing the tests for several reasons: high melting temperature (1455 °C), good thermal conductivity, good linear expansion coefficient, and high mechanical resistance. However, Ni electrodeposition on SiO_2_ fiber can have some drawbacks concerning first its insulating properties and the final coating appearance. Preliminary electrodeposition evaluation is typically required to determine the suitable operating conditions in which the coating is compact, homogeneous, and defect-free. Such considerations on the coating aspect are due to the dominant hydrogen evolution reaction (HER) caused by low hydrogen overvoltage on Ni metal [25,26,27,28]. Therefore, the optimization of operating parameters is a crucial stage in order to obtain a suitable compromise between the effective Ni deposition and the good aspect of the coating, maintaining a constant radial growth of the deposit, which is important to have a uniform distribution of stresses along the whole coated sensor. The present work focuses on metallurgical problems related to electrodeposited Ni coating and its behavior at a high temperature. The optical characterization of the same coated sensor has been deeply described in a previous research work [29].

This paper presents the electrodeposition process used to produce a Ni coating for applications of FBGs at high temperatures and the investigation of the Ni coating evolution, under thermal cycling at increasing temperatures by analyzing grain growth and oxidation resistance, and the corresponding change in the grating’s reflected spectrum. 

## 2. Materials and Methods

### 2.1. Fiber Preparation for the Ni Electrodeposition

**Figure 1:**
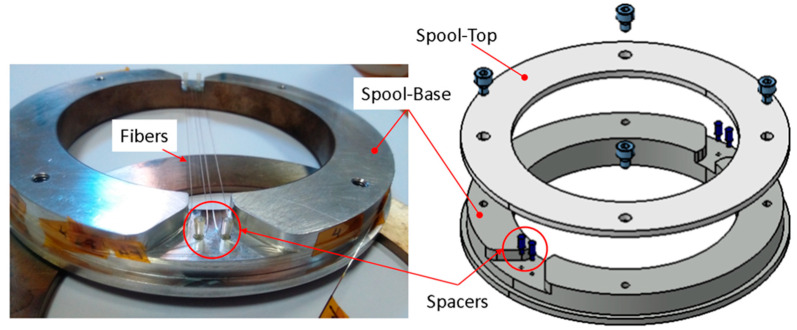
Fiber spool that is utilized for the gilding of the fiber segment via sputtering.

One of the most critical drawbacks in coating production is the metal electrodeposition on non-conductive surfaces, such as that of the SiO_2_ fiber. The whole surface should be conductive in order to permit reliable charge flow and effective metal reduction reaction during the electrodeposition process. Thus, this section describes the processing of fibers that are used for the electrodeposition of metal coatings, particularly gilding. Typically, 30 cm segments of a germanium-doped single-mode 9/125 optical fiber (9 µm in core and 125 µm in cladding diameters), without and with a grating, were prepared for testing. The excluding grating segments, called dummies, were used to optimize the electro-deposition process parameters, while the FBG segments, which have been cut by trying to keep the sensor in the middle of their length, were used to perform all the tests in which it is intended to follow the evolution of the spectrum, as the temperature varies. The thin layer of gold required to make the surface conductive was produced by means of sputtering, using the EDWARDS S150B sputter coater. The segment was first wound around a purposely designed aluminum spool, Figure 1.

The 9/125 fiber has an external 250-µm-acrylate coating to easily handle the fiber. The central section of each segment, which was centered within the spool aperture of 90 mm in diameter, was stripped (acrylate coating removal) to a length of 80 mm. In this way, the gold plating completely covered the portion of the stripped fiber.

**Figure 2:**
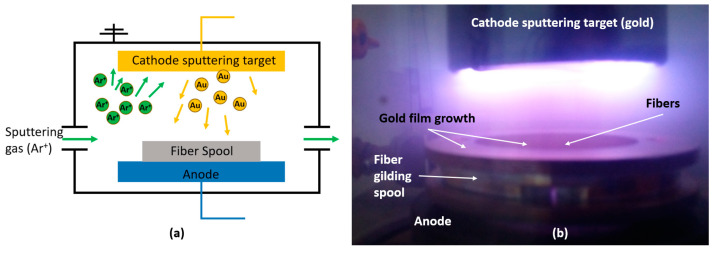
(**a**) Layout and (**b**) experimental picture of the sputter vacuum chamber operation.

The spool, capable of gilding 4 fiber segments simultaneously, was placed at the bottom of the sputter vacuum chamber, exposing the upper face to the sputter target, which was located on the top face of the vacuum chamber (Figure 2). The cycle involved two successive stages of sputtering lasting 5 min each. After the first step, the spool was rotated 180° to allow greater exposure of the fibers to the ion cloud and, consequently, to provide good uniformity and continuity in gold deposition to ensure conductivity of the fiber surface.

**Figure 3:**
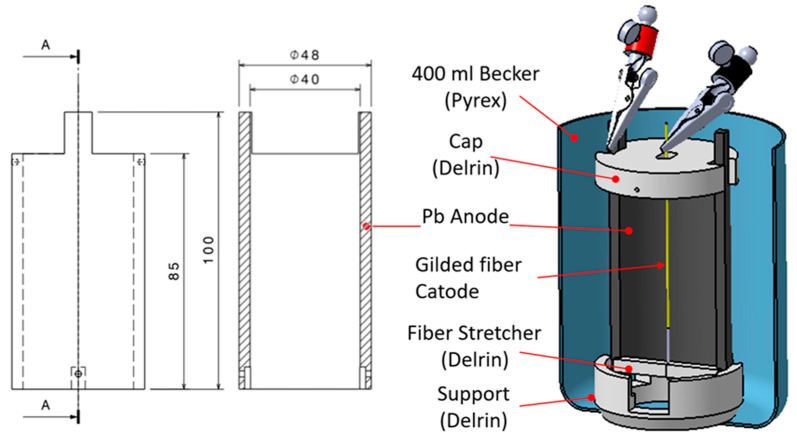
Cylindrical electrolytic cell used to perform the Ni coating via electrodeposition.

The gilded fiber segment was then inserted into the electrolytic cell consisting of a 400-mL Pyrex beaker glass vessel. To achieve Ni deposition on the fiber, the gold segment, working as a cathode, was inserted into the cylinder-shaped lead anode, and was held in place at the axis of the cylinder by the fiber stretcher, located at the bottom of the beaker, and by an alligator clip that gripped the fiber passing through the cap hole, Figure 3. 

### 2.2. Ni Coating via Electrodeposition

For the tests, 500 mL distilled water, 40 g/L nickel (II) sulfate hexahydrate (NiSO_4_·6H_2_O), and 20 g/L boric acid (H_3_BO_3_) were used to prepare the electrolyte. In order to carry out the Ni electrodeposition, the solution pH was adjusted by adding drops of sulfuric acid or caustic soda, reaching a pH of 4.8. The electrodeposition was performed by using a potentiostat/galvanostat Amel Instrument (model 2053) with a process duration of 180 min. After the electrodes were totally immersed in the bath at room temperature, the current flew at the electrode in order to have a 500 A/m^2^ current density (CD) for nickel electrodeposition. 

**Figure 4:**
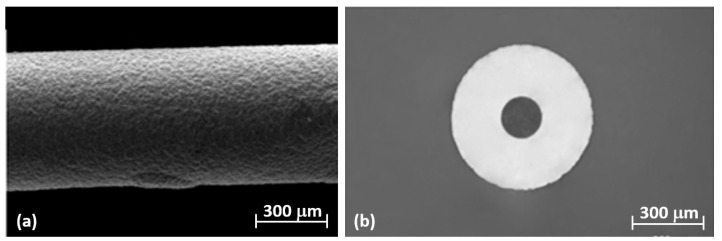
Micrographs of (**a**) superficial appearance and (**b**) cross section of Ni-coated fiber.

Deposit uniformity is a remarkable outcome that must be properly managed in order to avoid adverse effects on the signal and grating spectrum caused by anisotropic radial stresses of irregular deposits. Thus, the Ni-coated fiber was microscopically analyzed by using a scanning electron microscope (SEM). In Figure 4, the micrographs of the surface appearance and cross-section are visible. The SEM micrograph in Figure 4a of the Ni-coated fiber showed a uniform and compact morphology, while considering the cross-section, the coating was very regular, presenting a thickness of about 140 µm (Figure 4b). It is worth noting that typical surface defects (e.g., porosity, cracks, irregular growths, etc.) were not observed in the morphological analysis, which indicated that it was possible to obtain a well-distributed nickel coating around the fiber by means of proper selection and control of the operative conditions.

### 2.3. Coated Fiber Bragg Grating Samples

**Figure 5:**
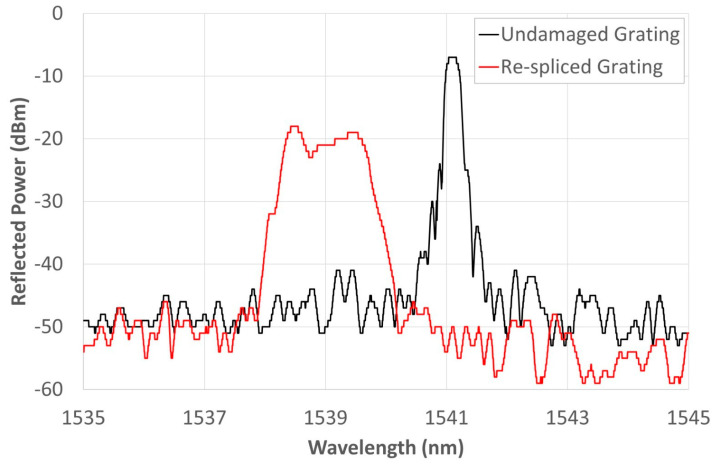
Comparative analysis of FBG sample underwent intentional breaking and subsequent re-splicing. The measurement was performed at RT and the CWL was identified at 1541.01 nm. Figure taken from reference [29].

**Figure 6:**
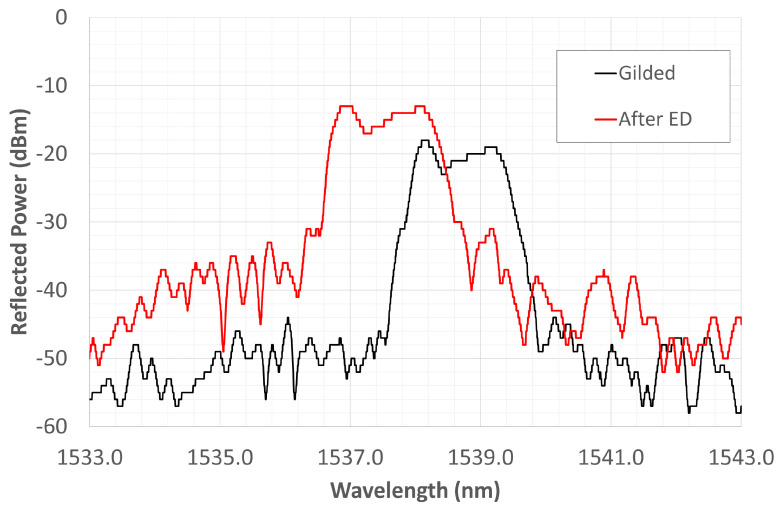
Spectrum evolution during gilding and after 24 h from the Ni electrodeposition.

For the present work, a set of FBGs, that had been cut and re-spliced along the grating, was tested. Such a procedure caused an obvious distortion of the original spectrum as shown in Figure 5. That result, which was taken from our previous paper [29], showed the possibility of regenerating the distorted spectrum by specific thermal cycling. Given that FBGs underwent cutting and re-splicing, the gratings’ spectra could suffer notable modifications in comparison with the undamaged one, as illustrated in Figure 5. In this figure, the black line represents the spectra of an undamaged grating, while the red line shows a reflected spectrum of the distorted grating by cutting/re-splicing procedure. Considering both spectra were obtained at RT, the CWL and full width at half-maximum (FWHM) were 1541.01 nm and about 0.555 nm, respectively. The spectrum has a well-defined peak that identifies the nominal wavelength of the FBG (i.e., the CWL). Qualitatively, the difference between the two spectra is obvious. The bell shape is retained but the base has widened, resulting in a less regular upper part that involves a range of wavelengths, rather than the CWL initially identified. The spliced segment passed through the gilding process and then the Ni electrodeposition. Figure 6 shows the change in the grating spectrum following two different steps of the process. The black curve shows the state of the spectrum after gilding as soon as the fiber is introduced into the beaker containing the electrolyte solution. The red curve was recorded 24 h after the end of Ni deposition. The spectrum has stabilized over a range of lower WLs than the initial value; there is also a small increase in the reflected power. The origin of the new spectrum shape is still under investigation. Rather than a plateau, it would appear to be the partial overlap of two very close spectra, with CWLs at 1538.44 nm (left end of the red curve in Figure 5) and 1539.535 nm (right end of the red curve in Figure 5), respectively. The splicing most likely caused an uncontrolled diffusion of the doping elements that led to an irregular alteration of the local refractive index, generating, the two gratings, and reducing their reflected power. Dopants diffusion is strongly temperature-dependent, and as temperature increases, as explained in [29], the reflected power of the overlapping portion decreases, accentuating the two end CWLs, which, at the same time, tend to move closer together until they merge into a single peak once 750 °C is reached. This would confirm that the cause of the new shape is due to the diffusive effect of the dopants, which, with increasing temperature, trend to re-diffuse in the SiO_2_ matrix.

### 2.4. Thermal and Cycling Treatments

Two different thermal treatments were performed in this section. The thermal treatments were performed by using a vertical muffle furnace, where a double coaxial alumina cannula, containing the FBG, was inserted and held from the furnace’s top. This arrangement, including a suitable closing with alumina cement, aimed to keep the internal temperature unchanged. Parallelly, a second tube with a K-type thermocouple read the temperature close to the grating in order to obtain a reliable reference value.

Firstly, the Ni-coated dummy sample was treated with two thermal treatments from RT up to 170 °C. Subsequently, each coated fiber was heated up to 800 °C using in both cases a heating rate of 2 °C/min (Table 1). It is well-known that during metal electrodeposition hydrogen can be absorbed on the metal surface due to HER. On the cathode, HER is easily recognized with the plentiful appearance of bubbles. In the case of Ni surface, its notable electrochemical properties as hydrogen catalysts make HER an expected phenomenon [30,31]. Accordingly, the dehydrogenation process of the electrodeposited nickel is a required stage in order to eliminate the remaining hydrogen and stress. Thereby, the thermal treatment was performed, as mentioned above and detailed in Table 1. Additionally, after the thermal treatment, the morphology of Ni-coated FBG was characterized by SEM micrographs, while characteristic crystallographic phases of the coated FBG were identified by using an X-ray diffraction (XRD) spectrometer equipped with a CuKα source.

Secondly, the FBG fiber, using a vertical muffle furnace, was also subjected to thermal cycling treatments from RT to 750 up to 800 °C; and cooling down to RT at 2 °C/min (Table 1). The thermal assessments started once the fiber’s grating reached equilibrium conditions (thermal and mechanical) for a duration of 15 h in order to hinder experimental interferences that could compromise the reliability of measurements.

## 3. Results and Discussion

The nickel electrodeposition tests were performed on both pre-gilded fiber and FBG sensor to ensure their conductivity. The need to carry out preliminary Ni electrodeposition tests on the fiber derives from the difficulty of obtaining a coating compact surface, due to the ease with which hydrogen develops during Ni electrodeposition. In fact, the parasitic HER can become predominant during the Ni coating, due to its low hydrogen overvoltage. Furthermore, the deposit should have a constant thickness and be smooth to guarantee a homogeneous distribution of the stresses on the whole sensor. The latter feature can be obtained by making the deposit grains as fine as possible. The minimization of both HER and grain size could be achieved by optimizing the process parameters (temperature, CD, electrolyte pH, and additives). In this study, temperature and CD effects have been considered, while the solution pH was 4.8 and boric acid was used as an additive, based on previous work [32].

### 3.1. Effect of Temperature on Grain Growth 

**Figure 7:**
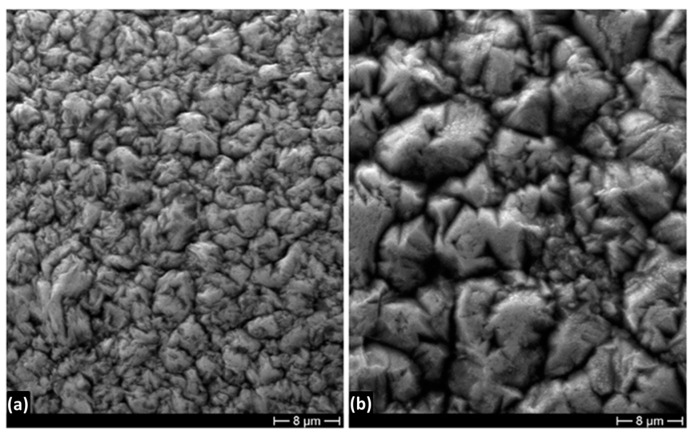
SEM micrographs showing the morphology of Ni deposits obtained at 250 A/m^2^ CD and (**a**) 20 °C and (**b**) 50 °C.

Two different temperatures, 20 °C and 50 °C, were tested for all the considered CDs. Particularly, Figure 7 shows the deposits obtained at 250 A/m^2^ CD. As can be seen, comparing Figure 7a,b, increasing the bath temperature from 20 °C to 50 °C the grain size increases, while the grain morphology remains unchanged, showing a pyramidal-like shape. It is generally expected that the grain size of the deposits increases by increasing the bath temperature because grain nucleus formation energy depends on the cathodic overpotential [33]. A large cathodic overpotential reduces the energy of nucleus formation, with a consequent increase in nucleus densities [34]. Consequently, since the cathodic overpotential decreases with increasing the bath temperature [35], it is expected that the deposit grain size increases. This is confirmed by experimental results.

### 3.2. Effect of Current Density on Grain Growth 

**Figure 8:**
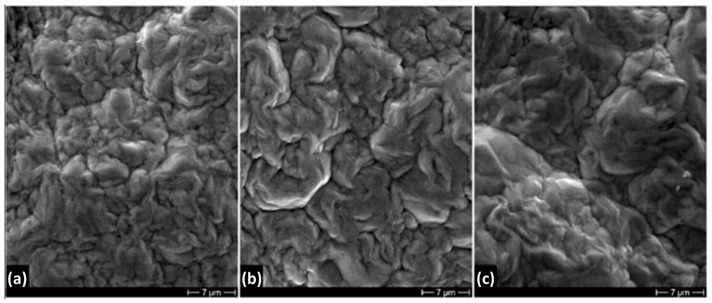
Morphology of deposits obtained at different current densities (**a**) 500 A/m^2^, (**b**) 750 A/m^2^ and (**c**) 1000 A/m^2^ at 20 °C.

The CD also plays an important role in the grain size of an electrodeposited coating [36]. In fact, an increase of CD leads to an increase in the cathodic overvoltage, and therefore to an increase in the nuclei density with a consequent decrease in the grain size of the deposit. Thus, a high CD could promote grain refinement [37,38]. The deposit micrographs reported in Figure 8a–c show an opposite behavior: the grain size increases with increasing the CD. The deposit of Figure 8c electrodeposited at the highest CD (1000 A/m^2^) presents the biggest grain size, that of Figure 8a electrodeposited at the lowest CD (500 A/m^2^) has the smaller grain size, while the deposit obtained at intermediate CD (750 A/m^2^) also shows an intermediate grain size. However, the effect of CD on the grain size of the Ni electrodeposited coating is still under discussion: among the different authors who have studied this effect, some of them reported a decrease in the grain size with CD [39,40], others an increase [39,41,42]. The behavior of our coating could be justified by the cylindrical symmetry of the cathode. In fact, increasing the deposit thickness by increasing time, the CD decreases, and that effect is more pronounced with higher CDs.

**Figure 9:**
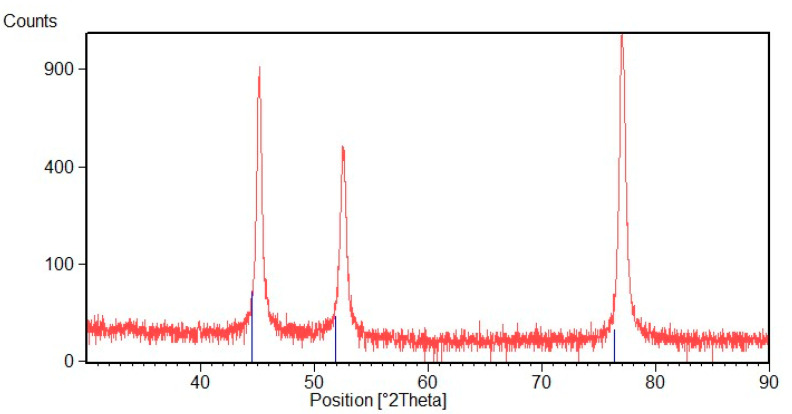
XRD diffraction pattern of Ni deposit.

In Figure 9, the XRD pattern of the Ni deposit is shown. By observing the figure, it is apparent that Ni peaks are slightly shifted toward higher 2θ value. This reveals that there is a contraction of the crystal lattice. In the Ni electrodeposition, this shift has been already reported in the literature, but it has not been explained [43]. This phenomenon could verify because, in the electrodeposition process, the lattice of the deposited metal must adapt to the metallic substrate, and the grain growth is affected by process parameters selected for the electrodeposition. The XRD pattern highlights also that deposited Ni is textured along a preferential [220] orientation.

**Figure 10:**
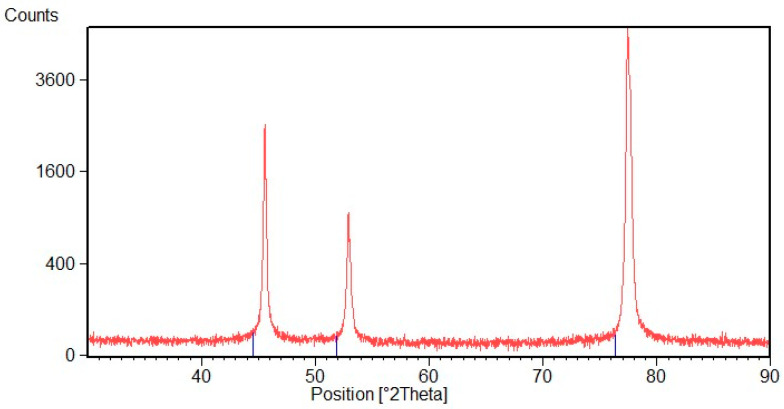
XRD diffraction pattern of Ni deposit after heat treatment at 170 °C.

**Figure 11:**
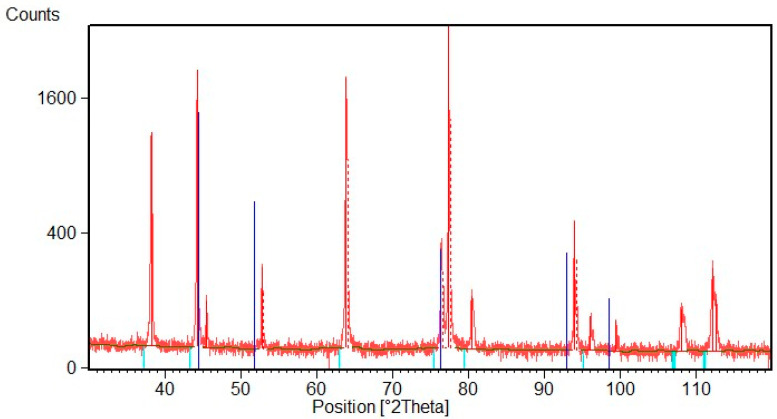
XRD diffraction pattern of Ni deposit after thermal treatment at 800 °C. The blue lines indicate Ni peaks, while light blue lines indicate NiO peaks of the standard patterns.

**Figure 12:**
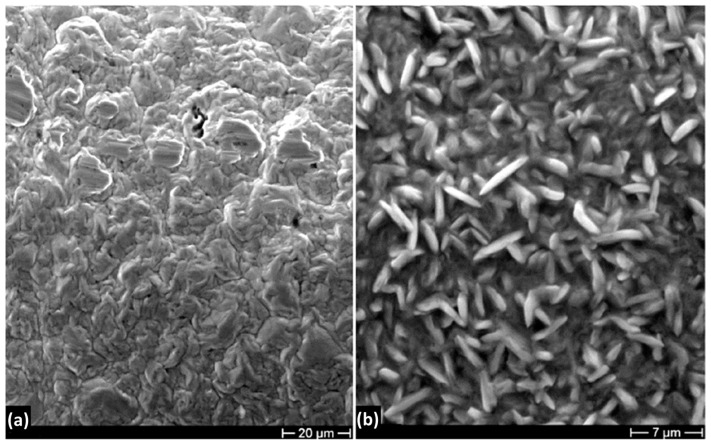
SEM micrographs of the Ni deposit after thermal treatment at (**a**) 170 °C and (**b**) 800 °C.

Figure 10 shows the XRD pattern of the Ni deposit after thermal treatment at 170 °C. It is possible to observe that this treatment does not seem to affect deposited Ni, and oxide peaks are not visible. Figure 11 shows the XRD pattern after thermal treatment at 800 °C. At this temperature Ni forms a NiO layer that, growing on the compressed Ni lattice, is characterized by a contracted crystal lattice. It is possible to note that both spectra are shifted toward higher 2θ value. SEM observations of the deposit surface reveal that after treatment at 170 °C there are no visible oxides on the metal surface, while after heat treatment at 800 °C the NiO layer is compact with an acicular morphology, Figure 12.

### 3.3. Effect of Thermal Treatment on FBG Spectrum 

**Figure 13:**
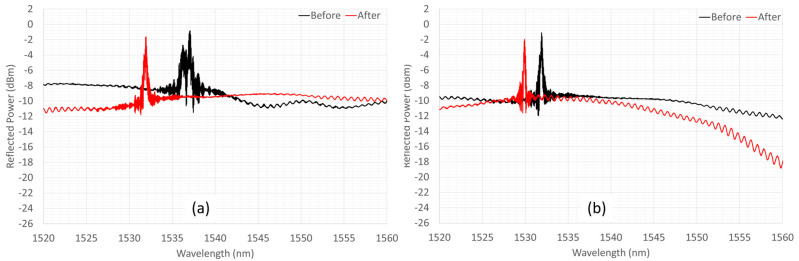
Grating spectrum evolution after heating-cooling cycling, from RT to: (**a**) 750 °C and (**b**) 800 °C.

**Figure 14:**
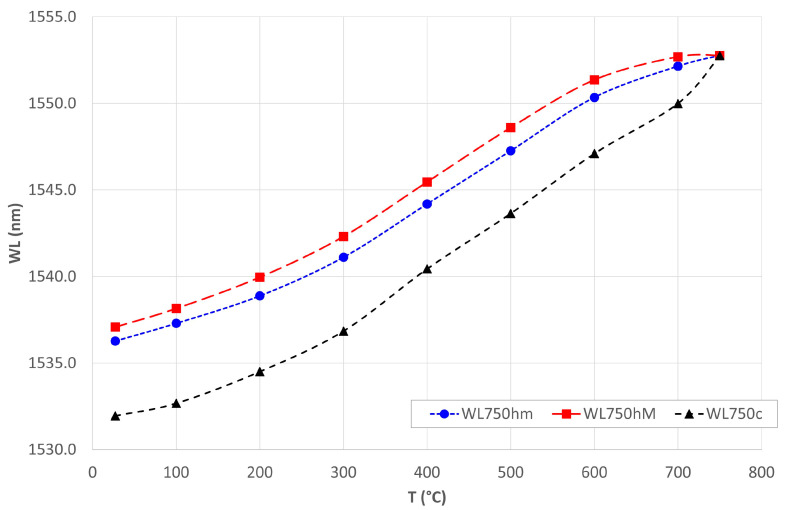
Spectrum’s peaks trend during thermal cycling.

As a result of the thermal cycling, the Bragg grating recovered the single-peak bell-like shape, as is shown in Figure 13. The black curve is the spectrum before the heating, the red one is the spectrum after the cooling cycle, from RT to 750 °C (Figure 13a), and from RT to 800 °C (Figure 13b). In the first two cycles, the spectrum maintains a very irregular shape, presenting 2 end peaks, rather than the typical CWL peak. After reaching 750 °C (Figure 13a), the spectrum rejoins the two peaks into a single CWL. The latter configuration remains stable in the cooling, until it returns to RT and in the next cycle, in which the Ni-coated piece of fiber is first heated to 800 °C and then cooled, again, to RT. The trend of the two parallel peaks and their rejoining into the single peak of the CWL is shown in Figure 14.

The blue and red curves indicate the two end-peaks’ WLs variation of the distorted spectrum, which move parallelly during heating from RT to 750 °C. Reaching 750 °C, the spectrum tends to narrow, bringing the peaks closer together (an effect clearly visible as early as 600 °C) until they join. The black curve indicates the cooling that returns the fiber to room temperature, in which case we see the change in WL of a single peak. The Ni coating protected the optical fiber and grating during the thermal cycle up to 800 °C, allowing regeneration of the spectrum. There remains to be investigated why the value of CWL at room temperature is much lower than the initial value. X-ray analysis showed an increase in the compressive state of the coating lattice structure due to the thermal cycling. This could also have a compressive effect on the grating that would justify the lower CWL, compared to the pre-heating state.

## 4. Conclusions

This work aimed both to determine the best operating parameters to obtain a nickel coating for optic fibers carrying FBG, which would be the most compact, adherent, and homogeneous, and to present the correlation between the morphology and structure obtained in the coating and the resulting alterations in the grating spectrum. The technique used was electrodeposition, which can guarantee greater stiffness, lower brittleness, and higher mechanical resistance. The coated grating, tested at high-temperatures up to 800 °C, showed stability after regeneration, despite the highly deformed spectrum caused by the re-splicing. The coating deposition and the subsequent heat treatments induce a final state of compression on the grating, well highlighted by the wavelength shift toward lower values during cooling. This compressed state is confirmed by the XRD spectra, which are shifted toward the right. This lattice compression is probably due to a variation of the lattice parameter. Temperature and CD selected for the electrodeposition process affect grain size, while thermal treatments produced grain growth and the formation of oxides well evidenced by the SEM and XRD analyses.

## Figures and Tables

**Table 1:** Operative conditions used during thermal treatment and thermal cycling.

Assessment	Heating Cycle (°C)	Cooling Cycle (°C)	Aim	Characterization
Thermal treatment on dummy samples	RT-170	170-RT	Elimination of hydrogen and stress	SEM and XRD
	RT-800	800-RT		
I Thermal cycle	RT-750	750-RT	Evaluation of high-temperature effect on coating	Grating analysis
II Thermal cycle	RT-800	800-RT		

## Data Availability

Not applicable.
